# Obesity and Weight Management in HIV: Epidemiology, Complications, and Emerging Treatments

**DOI:** 10.1007/s11904-026-00776-1

**Published:** 2026-02-24

**Authors:** Luke Pryke, John R. Koethe, Samuel Bailin

**Affiliations:** Division of Infectious Diseases, Department of Medicine, Vanderbilt University Medical Center, A-2200 MCN, 1161 21st Avenue South, Nashville, TN 37232 USA

**Keywords:** HIV, Weight gain, Antiretroviral therapy, Glucagon-like peptide-1 receptor agonist, Obesity, Weight management

## Abstract

**Purpose of Review:**

People living with HIV (PLWH) on contemporary antiretroviral therapy experience high rates of overweight/obesity, which predisposes to cardiometabolic disease and multiple other conditions with negative health consequences in this aging population. We aim to summarize the epidemiology and pathophysiology of obesity in PLWH and review recent advances in the therapeutic management of obesity.

**Recent Findings:**

The prevalence of overweight/obesity in PLWH mirrors long-standing trends in the general population. Obesity and weight gain have a complex, multifactorial pathogenesis and directly mediate detrimental metabolic changes that are common in PLWH. While lifestyle changes are important, surgical weight loss and recent advances in medical therapeutics are more effective at reducing obesity and obesity-related complications.

**Summary:**

Obesity in PLWH substantially increases the risk for cardiometabolic complications and poor health outcomes. Surgical and medical weight loss interventions are effective treatments to reduce obesity and obesity-related complications, though further research in PLWH is needed to define optimal management.

## Introduction

In the modern era, people living with HIV (PLWH) on antiretroviral therapy (ART) have longevity approaching that of people without HIV (PWoH). However, age-related cardiometabolic conditions have substantially increased, which occur earlier and with greater frequency than in PWoH [1, 2]. Excess adiposity is a major contributor to the development of cardiometabolic diseases, and predisposes to frailty, cognitive decline, some cancers, and several other conditions [3, 4]. PLWH have high rates of obesity [5, 6], which mirrors long-standing trends in PWoH [7]. HIV-specific risk factors also predispose to cardiometabolic conditions, including rapid weight gain after initiation of ART [8], residual HIV-related inflammation, dyslipidemia [9], and ectopic lipid deposition in skeletal muscle, liver, and other visceral sites [10]. Given the complex pathophysiology of obesity and its interaction with HIV-specific factors, effective interventions must address the multifaceted nature of obesity to reduce cardiometabolic conditions among individuals on long-term ART.

Lifestyle interventions, including dietary modifications and regular exercise, were effective in research studies [11] but are difficult to implement in the clinical setting, often hard to sustain, and have shown disappointing long-term outcomes [12, 13]. Interest in ART regimens as determinants of body weight, particularly with integrase strand transferase inhibitors (INSTIs) and tenofovir alafenamide (TAF), has led to ART-switch trials focused on weight loss and metabolic health [14]. However, recent data from randomized controlled trials suggest regimen changes to agents viewed as “weight neutral” or even “weight suppressive” have minimal impact, particularly among those with obesity [15, 16]. Surgical weight loss and emerging medical therapeutics including glucagon-like peptide-1 receptor agonist (GLP-1RA) and dual GLP-1 gastrointestinal inhibitory peptide receptor agonists (dual GLP-1 and GIP RAs) show great promise in not only reducing obesity but also improving obesity-related complications [17–19]. However, the timing of intervention in relation to ART initiation, how these interventions affect HIV-specific factors, and the implementation of these treatments in the clinical setting are an area of active research.

## Epidemiology of Weight Gain in People Living with HIV

Worldwide rates of individuals who are overweight or have obesity in the general population have increased in recent decades [20, 21], particularly in the US [22]. The epidemiology of obesity in PLWH has dramatically changed since the start of the HIV epidemic. Initially, HIV-associated wasting or weight loss in the context of low CD4 count was common in the era of few effective ART options and toxic metabolic side effects [23, 24]. This trend has largely reversed with the introduction of more effective and tolerable antiretrovirals, earlier treatment initiation, and a shared obesogenic environment with the general population in most resource rich and, increasingly, resource limited countries. Instead of a ‘return to health’ phenomenon, excessive weight gain among PLWH starting ART is now common and many individuals are overweight or have obesity at the time of HIV diagnosis [5, 6]. Those who initiate ART who are ≥ 60 years or have baseline BMI ≥ 30 kg/m^2^ experience larger weight gain [25, 26]. ART-specific weight changes are discussed in a separate section.

Compared with PWoH, obesity shows a sex-specific pattern in PLWH. Epidemiologic data across diverse populations consistently demonstrate that men living with HIV have lower body mass index (BMI) compared to men without HIV, whereas women living with HIV often have higher BMI compared to women without HIV [27, 28]. Data from the North American AIDS Cohort Collaboration on Research and Design (NA-ACCORD) found obesity rates at time of ART initiation increased from 11% between 1998 and 2000 to 17% between 2007 to 2010 [8]. Additionally, data from the Medical Monitoring Project and National Health and Nutrition Examination Survey found lower prevalence of obesity in men with HIV, but higher prevalence of obesity in women with HIV compared to the general population [27]. Data from outside of the US reveal a similar sex-specific pattern of obesity [, 30].

Additionally, a study examining more than 6000 patients from the HIV/AIDS Clinic Cohort Observational Database Project at the University of Alabama Burmingham from 2010 to 2011 found Black participants were more likely to have BMI ≥ 30 kg/m^2^, with 49% of Black women meeting the definition of obesity, compared to 24% of White women, 24% of Black men and 15% of White men [28].

In summary, obesity has emerged as a major health challenge in PLWH, which mirrors long-standing trends in PWoH [23, 31, 32] and disproportionately affects women and racial minorities.

## Detrimental Health Consequences of Excess Weight

Adipose tissue has a critical role in regulating systemic metabolic processes, through coordinated storage and release of triglycerides and secretion of beneficial hormones and cytokines [33]. However, when the storage capacity of adipose tissue is exceeded, this leads to adipocyte hypertrophy, tissue hypoxia and cellular stress, immune cell infiltration and tissue inflammation, and loss of beneficial adipokines that adversely affect systemic glucose and lipid metabolism [33]. Obesity has been causally implicated in the development of diabetes, cardiovascular disease, neurocognitive disease, and metabolic dysfunction associated steatotic liver disease (MASLD) [34–39]. Additionally, obesity is associated with reduced longevity. A recent Australian study in the general population evaluating 12,091 adults predicted that a 25 year-old man with obesity will lose between 8.3 and 10.4 years of life, while a 25 year-old woman will lose between 6.1 and 7.8 years compared to a 25 year-old without obesity [40]. PLWH with virologic suppression are at higher risk for developing cardiometabolic diseases compared to the general population, even after adjusting for BMI [38, 41, 42]. Many studies have implicated residual HIV-related inflammation that does not resolve with virologic suppression [43, 44]. Given the excess risk at baseline in PLWH, excess weight gain compounds the risk for cardiometabolic conditions in PLWH [8, 45] and this contributes to morbidity and diminished quality of life in this population [46, 47]. Obesity-related conditions including cardiovascular disease, malignancy and renal disease are now the leading causes of death in PLWH [48, 49]. Thus, interventions that reduce obesity-related conditions are critical for improving health outcomes in PLWH.

## HIV, Aging, and Ectopic Lipid Deposition

As a result of ART innovations and public health measures, the average age of PLWH has increased so that the single largest age group living with HIV in the United States is now 55–64 years of age [50]. Thus, the field is increasingly focused on healthy aging trajectories in PLWH. PLWH experience accentuated aging compared to PWoH and develop age-related cardiometabolic conditions earlier and at a higher rate [2, 51]. Aging is universally associated with loss of skeletal muscle mass and strength, and alterations in adipose tissue distribution with a shift from subcutaneous adpose tissue (SAT) storage towards visceral adipose tissue (VAT). In PLWH, HIV-specific factors (*HIV* per *se*, lipodystrophy, ART, rapid weight gain in ART-naive) along with obesity and aging shunt lipids away from SAT and towards VAT [52, 53]. Several studies have demonstrated cellular and molecular changes in SAT from PLWH might underlie changes in fat distribution [54–56]. Data from the German Heart Aging Study found PLWH had larger waist to hip ratio compared to PWoH controls despite a lower BMI, suggesting greater propensity to store lipids in VAT [30]. VAT size is strongly linked to insulin resistance (IR) and cardiovascular disease both in PLWH and in the general population [57–59]. Other ectopic areas including liver and skeletal muscle are equally deleterious [60–62]. PLWH develop myosteatosis at higher rates and this is an independent risk factor for IR [63]. Taken together, HIV-specific factors and obesity contribute to accentuated aging and early age-related cardiometabolic changes in PLWH though this remains an active area of investigation [61, 64].

## Obesity is Biologically Complex

Obesity is now understood to have a complex pathophysiology that spans genetic, biological, environmental, and psychosocial domains. Beyond a general increase in high calorie diets and sedentary lifestyles, obesity itself is thought to have a large heritable component estimated between 40–70% [65] as well as socioeconomic contributions [66] with higher rates of obesity among those experiencing food insecurity [67, 68]. This complex pathophysiology may in part explain why dietary and lifestyle interventions have disappointing results. A review of individuals from the National Weight Control Registry predicted that of those with obesity who lost 10% of their initial body weight through dietary changes and physical exercise, nearly 80% would regain the weight by one year [69, 70]. Caloric restricted weight loss results in compensatory mechanisms that increase appetite and alter energy utilization and storage, which favor weight gain. This process is mediated by a complex neurohormonal axis with decreases in anorectic hormones including peptide YY3-36, cholecystokinin, and amylin and concomitant increases in the orexigenic hormones ghrelin, GIP and pancreatic polypeptides [71]. Additionally, these hormonal adaptations to weight loss can persist, well beyond the initial caloric restriction period [72]. Ultimately, these physiologic changes are thought to underlie frequent weight increases after initial weight loss.

Traditional medical interventions for weight loss do not effectively alter these compensatory hormonal adaptions, which explains their modest weight loss effect. In contrast, GLP-1RA and dual GLP-1 and GIP RAs both promote weight loss and attenuate compensatory hormonal adaptations [73]. These agents and newer classes in early clinical development may change the paradigm of weight management that is critical for reducing weight-associated conditions [74], which contribute to increased healthcare costs and substantial morbidity and mortality [39, 75].

Taken altogether, weight gain and weight maintenance have many determinants, which in part explain why lifestyle changes alone are often not effective long-term. Recently approved weight-loss therapeutics show substantial promise in reducing obesity and weight-associated conditions.

## Weight Loss Interventions

### Incretin Analogs, GLP-1, GIP Receptor Agonists

GLP-1RAs and dual GLP-1 and GIP RAs constitute a novel therapeutic class that demonstrates high weight loss efficacy [17, 18, 76, 77]. Currently three drugs in this class have been approved in the US and internationally for weight loss: semaglutide, liraglutide and tirzepatide [78–80]. In addition, the SELECT trial demonstrated that semaglutide reduced major adverse cardiovascular events by 20% [81] and major kidney disease events by 22% [82]. Compared with semaglutide, tirzepatide had greater overall weight loss [83], and improves obstructive sleep apnea related outcomes [84]. Overall side effects in this class are generally mild, and predominantly gastrointestinal including nausea, diarrhea, vomiting and constipation [17, 18].

Thus far, data on outcomes in PLWH are limited, though several clinical trials enrolling PLWH have shown similar efficacy and safety profile of GLP-1RAs (Table 1). Eckard et al. enrolled 108 PLWH with lipohypertrophy who were randomized 1:1 to receive either semaglutide 1 mg weekly or placebo for 32 weeks (with an 8 week titration phase in the intervention arm) and found participants taking semaglutide had greater loss in total body weight, BMI, trunk fat, SAT, VAT, and liver fat [85]. The single-arm ACTG A5371 “SLIM LIVER” study enrolled PLWH with ≥ 5% intra-hepatic triglyceride (IHTG) for a 24-week treatment course with semaglutide 1 mg weekly, with a 4 week ramp-up period, and measured magnetic resonance imaging-proton density fat fraction (MRI-PDFF) of the liver. At 24 weeks, 29% of participants had complete MASLD resolution (IHTG < 5%) and 58% had ≥ 30% relative reduction in IHTG at the end of the study [86]. Of note, participants in these studies received semaglutide below those approved by the FDA for the treatment of obesity, which are either 1.7 mg or 2.4 mg once weekly during the maintenance phase of treatment. A few real-word retrospective studies have also found weight loss and antidiabetic effects in PLWH [87, 88].

**Table 1:** Comparison of studies examining effects of GLP-1RAs and dual GLP-1 and GIP RAs in PLWH.

Author	Haidar, et al.	Eckard, et al.	Nguyen, et al.	Lake, et al.
Participants Receiving Agent (ITT)	222	54	225	51
Primary outcome	Body weight change at 1 year	Changes at 32 weeks in adipose tissue quantity by body compartment	Change in weight on GLP-1RA therapy	Change in IHTG at 24 weeks
Weight loss, kg	6.5	6	5.4	7.8
Fat distribution changes	N/A	Decrease in total body fat, trunk, and abdominal fat including VAT and SAT, limb, pericardial, and liver fat. No statistically significant decrease in lean muscle mass	N/A	29% of patients with complete resolution of MASLD (IHTG < 5%), 58% had ≥ 30% reduction in IHTG
Time course (months)	12	8	13	6

In PWoH receiving GLP-1RA, studies have shown loss of lean muscle mass [89, 90]. PLWH at baseline have a heightened risk of sarcopenia compared to PWoH as discussed in a previous Section [61, 91]. In A5371, psoas muscle volume decreased by 9.3% over 24 weeks, but reassuringly, functional measures remained stable or improved [92]. Long-term cardiovascular and renal outcomes in PLWH are not well studied. A recent analysis of 1044 adults (63% male) with HIV and heart failure presenting to clinics in New York between 2017 and 2022 found a 46% decrease in overall mortality in those receiving GLP-1RAs (*p* = 0.04) [93]. However, as treatment was not randomly assigned in this observational cohort, potential confounding by indication, selection bias, and unmeasured disease severity may have influenced the findings. Larger randomized controlled trials are needed to establish the impact of GLP-1RA on mortality in individuals with HIV and heart failure.

Limited access to GLP-1RAs in the clinical setting due to nationwide shortages, relatively high drug prices, and variable insurance coverage remains an ongoing barrier to their therapeutic use [94]. Additionally, currently available GLP-1RAs must be used long-term, though long-term outcomes are lacking. Several landmark studies have shown substantial weight gain and regression of cardiometabolic improvements soon after stopping treatment in most participants [95, 96]. In a review of 125,474 adults who initiated GLP-1RA therapy between 2018 and 2023, discontinuation of therapy over 1 year was 53.6%, and over 2 years 72.2% owing to high cost, variable insurance coverage, drug availability, and side effect profile [97].

In summary, in PWoH GLP-1RA and dual GLP-1 and GIP RAs are highly effective and reduce weight-associated cardiometabolic diseases, which is a major treatment goal. Based on a few small studies, GLP-1RA treatment is likely safe and effective in PLWH, though outcome data for cardiometabolic conditions and whether these agents promote or exacerbate sarcopenia is an ongoing area of research.

### Growth Hormone-Releasing Factor Analogs

VAT size is tightly linked to cardiometabolic diseases [57]. Tesamorelin is a synthetic growth hormone-releasing hormone that increases endogenous growth hormone pulsatility, which has pleiotropic effects on metabolism including anabolic effects in muscle and catabolic effects in fat. In a study of 404 PLWH (> 80% virally suppressed) with abdominal lipodystrophy who were randomized to receive either tesamorelin or placebo over 12 months, those receiving tesamorelin had a 10.9% reduction in VAT seen on abdominal CT compared to 0.6% of those in the control arm [98]. Trunk fat decreased (mean 1 kg, via Dual-energy X-ray absorptiometry) as well as waist circumference and waist to hip ratio without a significant change in BMI, or limb or abdominal subcutaneous fat compartments. A secondary analysis of 341 PLWH found that over 26 weeks of treatment, administration of tesamorelin was associated with an increase in muscle density, as well as increase in total muscle area in the rectus and psoas regions [99]. Additionally, a recent study of 61 PLWH treated with tesamorelin for 12 months showed the treatment arm had a 37% relative reduction in hepatic fat fraction, suggesting that in addition to visceral obesity, tesamorelin might be an effective treatment for MASLD [100].

Tesamorelin’s clinical use is limited by the need for daily injections, re-accumulation of VAT upon discontinuation, its overall weight neutral effects, lack of long-term cardiovascular safety data, and limited data that it reduces the risk for cardiometabolic conditions [101–103].

### ART Regimen Changes

In recent years, newer ART regimens, particularly INSTI and TAF-based therapies, have received considerable attention regarding potential weight gain or weight-promoting effects. INSTI-based regimens have become standard of care owing to a high barrier to resistance and few side effects, and TAF has also replaced tenofovir disoproxil fumarate (TDF) in many practice settings, given concern for renal and bone toxicity among those receiving the latter agent.

The question of whether INSTIs and TAF caused substantial, clinically relevant weight gain in ART-naïve individuals came to wide attention with the prospective, 1053-participant ADVANCE trial in South Africa. In ADVANCE, treatment-naïve women randomized to dolutegravir (DTG) plus TAF/emtricitabine(FTC) gained an average of 10 kg at 96 weeks of treatment (representing an average 16% weight gain), compared to 5 kg for those receiving DTG plus TDF/FTC [104]; weight gains for men were similarly stratified by regimen but the differences were smaller. Furthermore, participants receiving the TAF-containing regimen had the highest risk of incident obesity and hypertension at 192 weeks of treatment [105]. Subsequent analyses, however, assessed CYP2B6 single nucleotide polymorphisms known to alter efavirenz (EFV) metabolism and showed “rapid metabolizers” of EFV had weight gain similar to those receiving DTG + TDF/FTC [106]. This finding suggested individuals with higher EFV plasma levels (i.e., those in the slower EFV metabolism categories) potentially experienced exposure-dependent weight attenuating effects from EFV. Data from pre-exposure prophylaxis (PrEP) studies strongly suggest TDF is also weight attenuating [107–110].

Several observational studies and secondary analyses of clinical trials also examined weight gain among treatment-naïve individuals initiating first regimens. One of the largest retrospective observational studies of weight gain among 22,972 treatment-naïve PLWH enrolled in the NA-ACCORD found that weight gain was greater with an INSTI-based regimen compared to a protease inhibitor- (PI) or non-nucleoside reverse transcriptase inhibitor- (NNRTI) based regimen [111]. Among INSTI drugs, weight gain was higher with DTG compared to raltegravir (RAL) and elvitegravir (EVG; co-formulated with the boosting agent cobicistat). Two notable limitations of this study were the low number of participants on TAF (none received bictegravir [BIC]), the large proportion of EFV-containing regimens in the NNRTI arm, and the lack of adjustment for the NRTI “backbone”. A subsequent analysis of 5680 participants enrolled in 8 ART comparative trials conducted between 2003 and 2015 found increased weight gain at 96 weeks in those initiating BIC, DTG or TAF vs. older regimens [112], further suggesting INSTIs and TAF may have differential effects on weight gain.

The variability in early weight gain after treatment initiation by regimen components raised the intriguing possibility that changes in ART, specifically a switch off of an INSTI or TAF, could induce weight loss (or weight stabilization) and improve metabolic health, particularly among women, Black people, and other subgroups at highest risk of weight gain and obesity. The DEFINE study was the first prospective ART switch trial specifically focused on weight and metabolism [14]. DEFINE randomized adults on an INSTI + TAF/FTC regimen with sustained viral suppression and history of a ≥ 10% weight gain in the prior 3 years to switch to darunavir/cobicistat/FTC/TAF or continue the current regimen. At 24 weeks, there was no significant difference in weight between the arms, suggesting the switch from an INSTI to a boosted PI (both with TAF/FTC) is not an effective weight loss strategy. The subsequent ACTG A5391 open-label, randomized trial switched PLWH with obesity (BMI *≥* 30 kg/m^2^) on a stable INSTI + TAF/FTC regimen to doravirine (DOR, a newer NNRTI), with or without a concomitant change from TAF/FTC to TDF/FTC. At 48 weeks there was no significant difference in weight in either the DOR + TAF/FTC or DOR + TDF/FTC arms versus those remaining on an INSTI + TAF/FTC regimen. Women and Black participants, two groups at higher risk of weight gain in prior studies, each comprised approximately 50% of the study cohort and did not derive significant benefit from a regimen change, nor did those with a history of substantial (> 10%) weight gain in the first three years of INSTI use. Based on the lower confidence interval limit of weight change within each arm, neither DOR arm resulted in *≥* 5% weight loss, indicating that a switch to an NNRTI-based regimen, with or without TDF, is not an effective strategy for weight reduction in PLWH with obesity.

Additional ART switch data comes from participants in the TANGO trial who were on a 3 drug regimen containing TAF and either remained on their pretrial regimen or switched to DTG/lamivudine (3TC). At 144 weeks, weight gain was not significantly different among those who stopped versus continued TAF (2.2 kg on DTG/3TC vs. 1.7 kg in those who remained on TAF) [15]. A study examining switch to cabotegravir and rilpivirine from BIC/FTC/TAF found no statistically significant difference in weight at 12 months [113]. A similar study which investigated a switch from BIC/FTC/TAF to islatravir/doravirine did not find significant weight changes at 48 weeks [114]. Consistently, a switch off of newer INSTIs or TAF has not been found to induce significant weight loss.

Overall, the evidence from PrEP, ART initiation, and switch trial data suggests TDF and EFV have weight-suppressing properties, while INSTIs and TAF appear to be largely weight neutral (Table 2). As such, the present data do not support switching ART in PLWH with overweight or obesity for the purpose of weight loss.

**Table 2:**
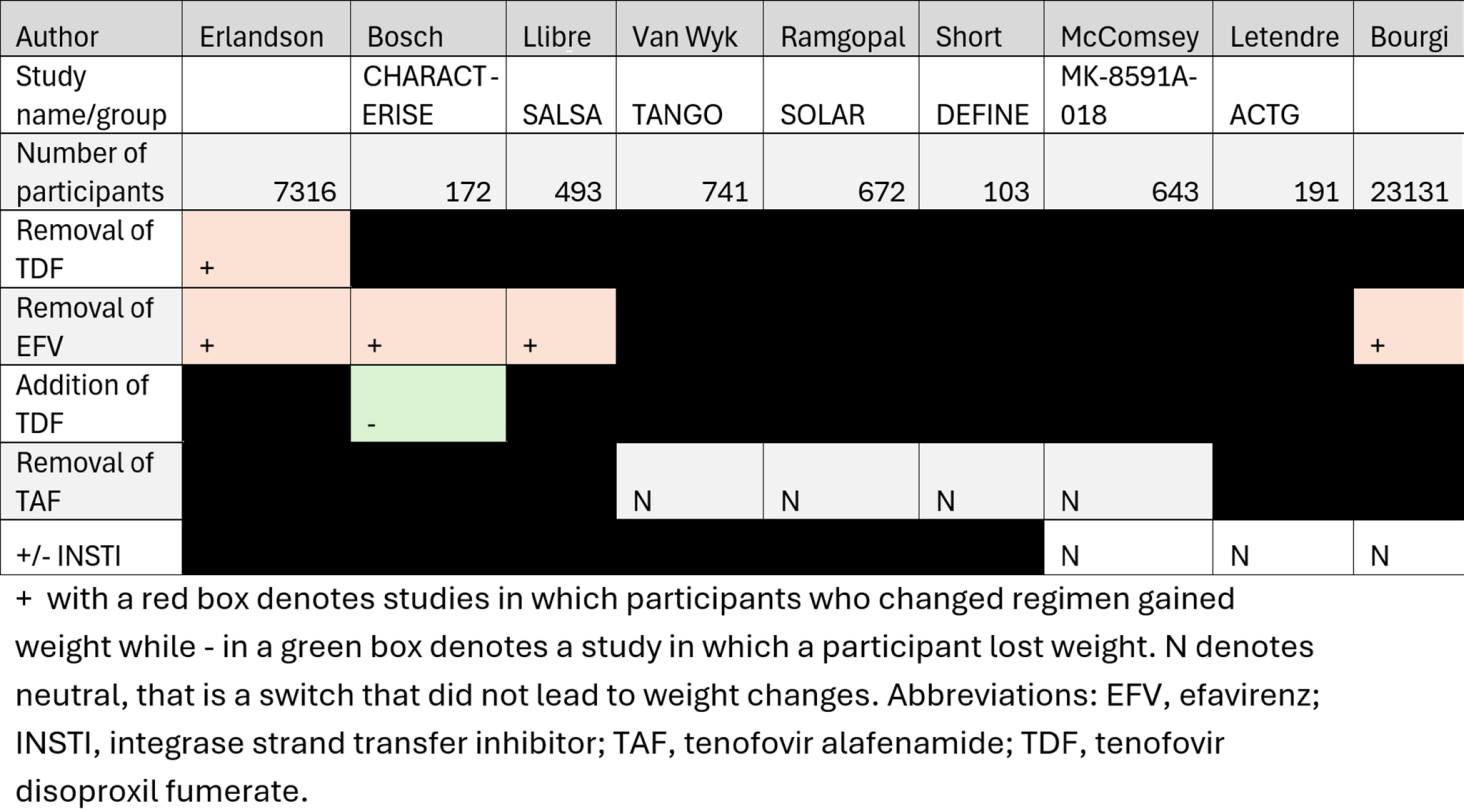
Comparison of studies examining antiretroviral therapy regimen switching and weight changes in PLWH

### Surgical Intervention

Bariatric surgery has long term outcome data showing sustained weight loss and improved life expectancy in people with obesity in the general population [115–117]. In PLWH, both Roux-en-Y gastric bypass (RYGB) and laparoscopic sleeve gastrectomy (LSG) are safe and effective for significant and sustained weight loss [118]. Both RYGB and LSG have unique effects on gut hormones, including differing effects on ghrelin and fasting peptide YY, with unclear weight loss implications [119]. RYGB in particular is associated with increased post prandial GLP-1 levels [120] as well as anti-inflammatory alterations in the gut microbiome [121]. A recent retrospective review of 10 PLWH who underwent bariatric surgery found greater weight loss at one year in patients receiving RYGB (25%) or LSG (17%) compared to laparoscopic adjustable gastric banding procedures (LAGB) (12%), which was sustained at 5 years [122]. Additionally, all patients remained virally suppressed. A retrospective analysis of 51 PLWH who received either RYGB or LSG between 2004 and 2022 in the Netherlands found a median 27% weight loss at 18 months [29]. All patients except for one remained virally suppressed post-surgery. Reassuringly, antiretroviral drug plasma concentrations post-surgery were within the therapeutic range except for boosted darunavir/ritonavir which was below the minimum effective concentration in a third of samples. In summary, bariatric surgery is safe and effective in PLWH and might be an attractive alternative to GLP-1RA therapy, which requires long-term use and may not be covered through insurance. Many bariatric surgery programs require surgical candidates to commit to dietary and lifestyle changes before pursuing weight loss surgery, which may be a barrier to some patients.

### Lifestyle Modification

For many years, diet and exercise were the primary and sole recommendations to patients for weight loss. Increased physical activity without dietary modification has been linked to lower obesity rates [123] and exercise itself without weight loss has been associated with reduced VAT [124]. In PLWH, studies that have evaluated specific dietary programs including initiation of the Mediterranean diet and low fat diet without caloric restriction did not show meaningful weight loss [125, 126]. Interventions that included caloric restriction and an exercise regimen were generally more successful. In one such study, women living with HIV (*n* = 18) who completed a 12-week intervention with nutrition sessions, a low calorie diet and 180 minutes of unsupervised exercise lost an average of 7.3% total body weight [127]. Another 12-week study (*n* = 40) which similarly included caloric restriction and exercise in its intervention arm with male and female participants found 4.5% weight loss at the end of the study [128]. A 2017 study designed to assess the metabolic effects of weight loss enrolled women (*n* = 28) with and without HIV in a program that included calorie restriction through meal replacements and found that weight loss was similar between groups (−8 kg) but fat loss was more pronounced in women without HIV (−7.1 kg) compared to women with HIV (−5.8 kg). Women with HIV on average required six additional weeks to meet weight loss goals (18 vs. 12 weeks). Despite longer time requirements for weight loss in those with HIV, reassuringly, participants who followed the meal plans were found to have similar improvements in metabolic health in both groups [129]. Another recent study enrolled PLWH (*n* = 28) who were educated about the DASH diet and instructed to exercise 10,000 steps daily. Participants lost a mean 4.6% body fat over six months [130]. In contrast to the above studies that combined caloric restriction and exercise to promote weight loss, a recent study enrolled both PLWH and PWoH to participate in 24 high-intensity interval training sessions over 8 weeks and found no significant difference in body composition at the end of the study. However participants had decreased IR and serum insulin levels, which was more pronounced in PLWH [131]. These studies show that combined dietary and exercise interventions in PLWH can result in modest weight loss and improved metabolic measures. Diet and exercise have an important adjunctive role with surgical weight loss and medical therapeutics but are less effective and less likely to promote long-term weight loss when used alone in the clinical setting.

### Future Directions

To date, the vast majority of studies on weight management and use of GLP-1RA are from the general population. While GLP-1RA reduce the risk for several cardiometabolic conditions in the general population, it is not known yet whether this extends to PLWH beyond hepatic steatosis and type 2 diabetes mellitus. PLWH are at increased risk for cardiometabolic conditions so it is possible PLWH may have greater benefit than the general population, though future studies are necessary to explore this. Additionally, multiple GLP-1RAs and a dual GLP-1 and GIP RA are approved for weight loss with more in preclinical trials, so comparative trials are necessary to understand which medications would provide the most benefits. Important end points should include weight loss, change in body composition, markers of IR and lipids that might predispose patients to cardiometabolic complications, change in lean mass, and muscle and liver density among other factors.

While the results from ACTG A5371 are reassuring that GLP-1RA do not adversely affect skeletal muscle function, larger studies will be needed to evaluate their influence on both muscle mass and muscle function. Finally, the optimal timing of these weight loss interventions is not known. Weight gain is greatest within the first two years of ART initiation and is the time at which the risk for cardiometabolic conditions such as insulin resistance emerge. Whether weight loss medications should be initiated during this period and if so, at what point, has not been defined to date. With newer oral incretins coming onto the market, trials examining broader use in PLWH will be more feasible.

## Conclusions

As rates of obesity rise and PLWH age, age- and obesity-related cardiometabolic diseases are increasingly common and contribute to substantial morbidity and mortality. Thus, new approaches that not only treat obesity but also improve and prevent obesity-related complications are urgently needed. While there has been substantial interest in changing ART regimens to weight neutral or weight suppressing therapy, randomized controlled switch trials have shown this strategy is ineffective. In contrast, GLP-1RA are changing the paradigm of weight management, and increasingly show efficacy in preventing and treating a wide range of conditions that affect PLWH. While most data are from the general population, a few clinical trials and retrospective studies in PLWH suggest GLP-1RA are both effective and safe. However, future studies are needed to clarify the optimal timing for their use in relation to ART initiation, and whether use in PLWH who do not have a current indication may derive benefit. Additionally, clinical use to date has been limited by high cost and variable insurance coverage. Surgical weight loss is another effective and safe option in PLWH with better data on long-term outcomes. Surgical weight loss and recent innovations in therapeutics are critical tools to improve health outcomes in PLWH.

## Key References

- Eckard AR, Wu Q, Sattar A, et al. Once-weekly semaglutide in people with HIV-associated lipohypertrophy: a randomised, double-blind, placebo-controlled phase 2b single-centre clinical trial. Lancet Diabetes Endocrinol. Aug 2024;12(8):523-534. 10.1016/s2213-8587(24)00150-5.
  - ○ Randomized controlled trial of PLWH (n=108) receiving semaglutide 1mg weekly over 32 weeks which found significant decrease in total body weight (10.4%) and total body fat (18.9%) without increased adverse events.
- Lake JE, Kitch DW, Kantor A, et al. The Effect of Open-Label Semaglutide on Metabolic Dysfunction-Associated Steatotic Liver Disease in People With HIV. Ann Intern Med. Jun 2024;177(6):835-838. 10.7326/M23-3354.
  - ○ Single-arm open-label trial of semaglutide 1mg weekly among PLWH (n=51). 58% of participants saw ≥30% reduction in IHTG, with 29% having complete resolution of MASLD (<5% IHTG) over the study period.
- Erlandson KM, Carter CC, Melbourne K, et al. Weight Change Following Antiretroviral Therapy Switch in People With Viral Suppression: Pooled Data from Randomized Clinical Trials. Clin Infect Dis. Oct 20 2021;73(8):1440-1451. 10.1093/cid/ciab444.
  - ○ Pooled analysis of PLWH (n=7316) who were either randomized to switch ART or remain on current therapy. Weight gain from initiation plateaued by 48 weeks, largest gains seen in those switching off TDF or EFV.
- Anderson D, Ramgopal M, Hagins DP, et al. DEFINE: A Prospective, Randomized, Phase 4 Trial to Assess a Protease Inhibitor-Based Regimen Switch Strategy to Manage Integrase Inhibitor-Related Weight Gain. Clin Infect Dis. Mar 17 2025;80(3):602-612. 10.1093/cid/ciae449.
  - ○ Randomized open label trial that investigated switch from INSTI to PI based ART regimen in PLWH (n=103). There was no significant change in body weight at 24 weeks.
- Zino L, Wit F, Rokx C, et al. Outcomes of Bariatric Surgery in People With Human Immunodeficiency Virus: A Retrospective Analysis From the ATHENA Cohort. Clin Infect Dis. Nov 30 2023;77(11):1561-1568.10.1093/cid/ciad404.
  - ○ Retrospective analysis of PLWH (n=51) who underwent bariatric surgery. 85% of patients had >20% total body weight loss at 18 months without virologic failure.

## Data Availability

No datasets were generated or analysed during the current study.
